# Neural network interpretation using descrambler groups

**DOI:** 10.1073/pnas.2016917118

**Published:** 2021-01-26

**Authors:** Jake L. Amey, Jake Keeley, Tajwar Choudhury, Ilya Kuprov

**Affiliations:** ^a^School of Chemistry, University of Southampton, Southampton SO17 1BJ, United Kingdom

**Keywords:** machine learning, interpretability, digital signal processing, electron spin resonance

## Abstract

The lack of interpretability and trust is a much-criticized feature of deep neural networks. In fully connected nets, the signaling between inner layers is scrambled because backpropagation training does not require perceptrons to be arranged in any particular order. The result is a black box; this problem is particularly severe in scientific computing and digital signal processing (DSP), where neural nets perform abstract mathematical transformations that do not reduce to features or concepts. We present here a group-theoretical procedure that attempts to bring inner-layer signaling into a human-readable form, the assumption being that this form exists and has identifiable and quantifiable features—for example, smoothness or locality. We applied the proposed method to DEERNet (a DSP network used in electron spin resonance) and managed to descramble it. We found considerable internal sophistication: the network spontaneously invents a bandpass filter, a notch filter, a frequency axis rescaling transformation, frequency-division multiplexing, group embedding, spectral filtering regularization, and a map from harmonic functions into Chebyshev polynomials—in 10 min of unattended training from a random initial guess.

## Significance

Artificial neural networks are famously opaque—it is often unclear how they work. In this communication, we propose a group-theoretical way of finding out. It reveals considerable internal sophistication, even in simple neural networks: our nets apparently invented an elegant digital filter, a regularized integral transform, and even Chebyshev polynomials. This is a step toward saving reductionism. For centuries, the philosophical approach to science has been to find fundamental laws that govern reality, to test those laws, and to use their predictive power. Black-box neural networks amount to blasphemy within that school, but they are irresistible because they “just work.” Explaining how they work is a notoriously difficult problem, to which this paper offers a partial solution.

Popular as the practice may be, simply training a neural net to perform a task, without giving an explanation of how it works, is increasingly frowned upon (1, 2)—neural network training is often just regression using the chain rule (3), and the resulting black box does not fit comfortably into the methodological framework (4, 5) of science and engineering. The concerns about deep neural nets are interpretability and trust, for which, at the moment, not even the definitions are settled. We can approximately define interpretability as *“*the possibility of finding out why and how it works” in the reductionist (4) and critical rationalist (5) sense, and trust as “rigorous quantification of uncertainties in the output.” Other related notions—intelligibility (6), algorithmic transparency (7), decomposability (6), attributability (8), transferability (9), and robustness (10)—may be viewed as aspects of those two general themes. Ultimately, the right answer for the right reasons is needed, accompanied by a measure of certainty (11).

A fully connected feed-forward artificial neural network with an input vector **x** and an output vector **y** is equivalent to the following function:

$$y=F_{n}W_{n}F_{n-1}W_{n-1}\cdots F_{1}W_{1}x,$$ [1]

where **W**_*k*_ are weight matrices, *F*_*k*_ are nonlinear activation functions, and bias vectors are not specified because, in this case, they are equivalent to having one extra input line. It is convenient to supply and receive arrays of input and output vectors; those will be denoted **X** and **Y**, respectively. The horizontal dimension of **W**_1_ is ordered in the same way as **x**; the vertical dimension of **W**_*n*_ is ordered in the same way as **y**; all other dimensions of **W**_*k*_ are not ordered because backpropagation training does not require them to be, and the initial guess is random. We call such weight matrices “scrambled”: they are two linear transformations—one from the left and one from the right—away from a representation with ordered input and output.

## Descrambler Group

We assume that neural networks are interpretable—that, for each layer *k*, a transformation **P** exists that brings the signal array *F*_*k*_**W**_*k*_ ⋅⋅⋅ *F*_1_**W**_1_**X** into a form that clarifies, to a competent human, the function of the preceding layers. We call this a “descrambling” transformation. The activation functions are not varied in the training process, and, therefore, this transformation must be applied to the weight matrices and judged on the output signals, for which some interpretability metric must be designed.

The transformation should be linear, so that linear combinations of signals are descrambled consistently. Information should not be lost, and therefore the transformation must be invertible. Transformations may be applied sequentially, and there exists a unit transformation that does nothing. That is the definition of a group which we will call the descrambler group. At the *k*^th^ layer of the network, it must be a subgroup of the general linear group of all automorphisms of a *d*_*k*_-dimensional vector space, where *d*_*k*_ is the output dimension of the layer. It should be a supergroup of the permutation group of *d*_*k*_ perceptrons within the layer, but should preferably be continuous and connected because discrete optimization is hard. Of those, *SO*(*d*_*k*_)—the connected group of all proper orthogonal transformations of a *d*_*k*_-dimensional vector space—is particularly promising, because physical signals are often defined up to an orthogonal transformation (e.g., cosine transform) and because the elements of *SO*(*d*_*k*_) are continuous and differentiable functions of a finite number of real parameters.

The act of wiretapping the network at a particular layer then consists of inserting a unit operator **P**^−1^**P** before or after a weight matrix:

$$\begin{array}{c} Y=F_{n}W_{n}\cdots F_{k}P^{-1}PW_{k}\cdots F_{1}W_{1}X\\ or\\ Y=F_{n}W_{n}\cdots P^{-1}PF_{k}W_{k}\cdots F_{1}W_{1}X \end{array},$$ [2]

and maximizing or minimizing such a function Λ of **PW**_*k*_ ⋅⋅⋅ *F*_1_**W**_1_**X** or **P***F*_*k*_**W**_*k*_ ⋅⋅⋅ *F*_1_**W**_1_**X**, as would quantify the features forming the basis of the interpretation, for example

$$P=\arg\left\{\begin{array}{l} \text{min}\\ \text{max} \end{array}\right\}\Lambda \left(PW_{k}\cdots F_{1}W_{1}X\right).$$ [3]

Much creativity may be needed to construct that function: it must take a signal and return a quantitative figure of merit for some problem- or domain-specific definition of “interpretable.” This could be a function involving measures of smoothness, periodicity, monotonicity, locality, autocorrelation, Shannon entropy, deviation from the expected statistics on luminosity and chromaticity, etc.

In our context—digital signal processing—the smoothness of the time-domain signal is a promising metric: a transformation that makes every intermediate signal across a large input library simultaneously smooth is also likely to make them physically meaningful. We have chosen Tikhonov smoothness—the squared Euclidean norm of the second derivative—as the metric to be minimized:

$$\Lambda \left(v\right)={\left\|Dv\right\|}^{2},$$ [4]

where **D** is a representation of the second derivative operator on a finite grid with *d*_*k*_ points; we use Fourier spectral differentiation matrices (12).

When multiple output vectors are concatenated into a matrix, the sum of squares of their Euclidean norms is the square of the Frobenius norm of that matrix. Therefore, applied to the output array of the *k*^th^ layer of the network, the Tikhonov smoothness criterion becomes:

$$P=\underset{P}{\arg\min}{\left\|DPW_{k}\cdots F_{1}W_{1}X\right\|}_{\text{F}}^{2},\;{\left\|A\right\|}_{\text{F}}^{2}=\text{Tr}\left(A^{\text{T}}A\right),$$ [5]

where ||_||_F_ denotes Frobenius norm, and **X** is a large enough array of input vectors (in practice, the entire training database). Importantly, Eq. **5** is not equivalent to smoothing the columns of the weight matrix by minimizing ${\left\|DPW_{k}\right\|}_{\text{F}}^{2}$. This is because only smoothness in the outgoing data is sought—a weaker requirement. Eq. **5** is also a weaker requirement than placing a Tikhonov penalty on the weight matrix at the training stage—an interpretable matrix need not itself be smooth, it only needs to produce intelligible signaling. Accordingly, the metrics being optimized in Eqs. **3** and **5** refer not to the weight matrices, but to the intermediate signal arrays.

In the absence of constraints, the obvious solution to Eq. **5** is **P** = 0—this is why a group-theoretical approach is needed, where **P** is generated by the Lie algebra of the descrambler group, and thus constrained to be nonsingular. However, the usual exponential map **P** = exp(**Q**) has expensive derivatives and numerical accuracy problems in finite precision arithmetic. We have therefore opted for a different connection between *SO*(*d*_*k*_) and its algebra, called Cayley transform (13):

$$P=\frac{1-Q}{1+Q},$$ [6]

where the numerator acts first, and **Q** is an antisymmetric matrix. Cayley transform is less sensitive to extreme eigenvalues than the matrix exponential. It is also easier to differentiate (*SI Appendix*, Section S1) with respect to **Q**. The general case remains as in Eq. **3**, for example

$$Q=\arg\left\{\begin{array}{l} \text{min}\\ \text{max} \end{array}\right\}\Lambda \left(\frac{1-Q}{1+Q}W_{k}\cdots F_{1}W_{1}X\right),$$ [7]

and the specific case of hoping for Tikhonov smoothness in the output of the weight matrix of a particular layer is equivalent to minimizing

$$\eta_{\text{T}}\left(Q\right)={\left\|D\frac{1-Q}{1+Q}W_{k}\cdots F_{1}W_{1}X\right\|}_{\text{F}}^{2},$$ [8]

with respect to the real antisymmetric matrix **Q**. The gradient ∂*η*_T_/∂**Q** is cheap (*SI Appendix*, Section S1), meaning that quasi-Newton optimizers like low-memory Broyden-Fletcher-Goldfarb-Shanno method (14) may be used. Memory utilization is likewise not a problem—Frobenius norm-square is additive with respect to the columns of **X**, which may be fed into the calculation one by one or in batches. Thus, if a network can be trained on some hardware, it can also be descrambled on the same hardware.

## Fredholm Solver Networks and DEERNet

Consider the trajectory *γ*(*x*,*t*) for a property *γ* in a quantum system with a parameter *x*. When the sample contains an ensemble of systems with a probability density *p*(*x*) in that parameter, the result Γ(*t*) of the ensemble average measurement is given by Fredholm’s integral (15):

Γ(t)=∫p(x)γ(x,t)dx, [9]

where *γ*(*t*,*x*) is sometimes called the “kernel”; its exact form depends on the physics of the problem. This integral is at the heart of applied quantum mechanics, used (directly or indirectly) for interpretation of any physical experiment by a model with distributed parameters. Given an experimentally measured Γ(*t*), extracting *p*(*x*) is hard: without regularization, this is an ill-posed problem (16), and regularization brings in a host of other complications (17). Deep neural networks perform unexpectedly well here (18), but no explanation exists as to why.

**Fig. 1.**
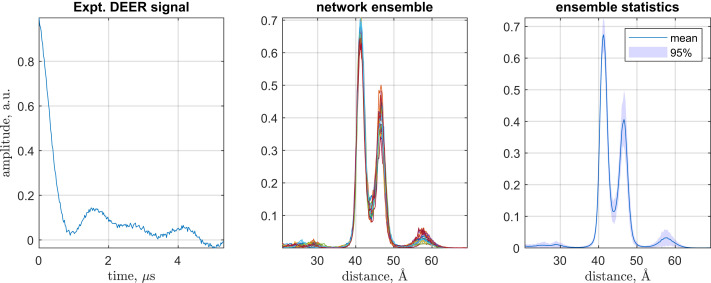
A typical DEER dataset from structural biology work, where distance measurement in biomolecules is often done by inserting magnetic tags and recording their dynamics under the action of the distance-dependent magnetic dipolar interaction (19). *Left* shows the electron spin echo modulation between two iodocateamido-PROXYL spin labels attached to the incoming cysteines in the V96C, I143C double mutant of Light Harvesting Complex II in *n*-octyl-β-d-glucoside micelles (33). *Center* shows distance probability densities returned by an ensemble of independently trained neural networks in DEERNet (18). *Right* contains statistics across the neural network ensemble. Expt., experimental; a.u., arbitrary units.

Our instance of this problem came from structural biology: molecular distance determination using double electron–electron resonance (DEER) (19). We generated a large database of realistic distance distributions and complications (noise, baseline, etc.) and converted them into what the corresponding experimental data would look like. Acting out of curiosity, we put together a fully connected feed-forward neural net and trained it to perform the inverse transformation—from noisy and distorted Γ(*t*) back into *p*(*x*). Because the problem is ill-posed, this was not supposed to be possible. The network did it anyway (Fig. 1) and matched the best regularization solver there is (18).

Mathematicians had looked at such things—neural network “surrogate” solutions to Fredholm equations had been attempted (20), and accuracy bounds are available (21). In 2013, Jafarian and Nia (22) proposed a feedback network built around a Taylor expansion of the solution; a feed-forward network proposition was published in 2012 by Effati and Buzhabadi (23). Both groups reported accurate solutions (22, 23), but neither looked at applications or asked the question about how a neural network actually manages to regularize the problem.

Given the precarious interpretability of quantum mechanics itself, demanding it from a neural network trained on quantum mechanics may seem unreasonable. However, this case is an exception: electron spin dynamics is very well understood, and the networks in question are uncommonly small—only 256 perceptrons wide, with at most 10 layers (18). We have therefore picked DEERNet as a test case for the descrambler group method. The simple and clear case involving two fully connected layers is discussed here; the case with three fully connected layers is in *SI Appendix*, section S4.

## Descrambling DEERNet

The simplest DEERNet has the following layer structure: vector input > fully connected > sigmoidal function > fully connected > logsig function > vector regression. The logsig activation function is necessary to ensure that the output (which has a physical meaning of probability density) stays positive. The input and the output are 256 elements wide, but the link dimension may be reduced to 80 by eliminating insignificant singular values (24) from the weight matrices of fully connected layers. The input dimension of **W**_1_ is time-ordered (Fig. 1, *Left*), and the output dimension of **W**_2_ is distance-ordered (Fig. 1, *Right*), but the link dimension connecting **W**_1_ and **W**_2_ is scrambled.

**Fig. 2.**
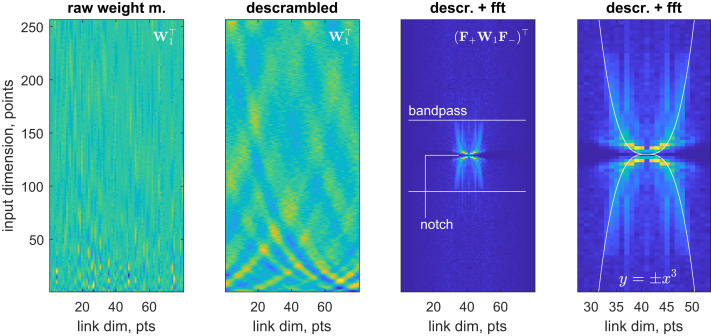
Spontaneous emergence of a sophisticated digital filter in the first fully connected layer of a DEERNet (18) neural network. From left to right: raw weight matrix of the input layer, descrambled weight matrix, symmetrized absolute value two-dimensional fast Fourier transform of the descrambled weight matrix, and a zoom into the central portion of that Fourier transform with a cubic curve overlaid. The layer applies a low-pass filter to remove high-frequency noise seen in the *Left* panel of Fig. 1; a notch filter at zero frequency to remove the nonoscillatory baseline; and also appears to be rearranging frequencies in such a way as to effectively take the cubic root of the frequency axis within the filter band—apparently, to account for the fact that the quantum beat frequency in DEER (19) is an inverse cubic function of the distance between the spins. Dim., dimension; m., matrix; fft., fast Fourier transform; descr. descrambled; pts., points.

Applying the descrambler group method to minimize the second derivative norm of the output of **W**_1_ (Fig. 2, leftmost panel) reveals a rich structure (Fig. 2, second panel from left)—the interlocking wave pattern indicates that some kind of frequency conversion is being performed on a signal that stays in the time domain. Inserting forward (**F**_+_) and backward (**F**_−_) Fourier transforms into the corresponding equation:

$$y=W_{1}x\;\Rightarrow \;F_{+}y=F_{+}W_{1}F_{-}F_{+}x,$$ [10]

demonstrates that the input signal frequency spectrum **F**_+_**x** is connected to the output signal frequency spectrum **F**_+_**y** by **F**_+_**W**_1_**F**_−_ matrix. Computing and plotting this matrix (Fig. 2, third image from left) reveals the function of the first fully connected layer: it applies a low-pass filter to eliminate high-frequency noise, a notch filter at the zero frequency to eliminate the nonoscillatory baseline, and performs frequency rearrangement in such a way as to effectively take the cubic root of the frequency axis within the filter band (Fig. 2, rightmost panel). The latter operation appears to reflect the fact that the quantum beat frequency in the kernel function of DEER depends on the cube of the distance (19):

$$\begin{array}{c} \gamma \left(r,t\right)=\sqrt{\frac{\pi }{6Dt}}\left[\cos\left[Dt\right]\text{FrC}\left[\sqrt{\frac{6Dt}{\pi }}\right]+\sin\left[Dt\right]\text{FrS}\left[\sqrt{\frac{6Dt}{\pi }}\right]\right]\\ D=\frac{\mu_{0}}{4\pi }\frac{\gamma_{1}\gamma_{2}\hbar }{r^{3}};\;\text{FrC}\left(x\right)=\int_{0}^{x}\cos\left(t^{2}\right)dt\;\text{FrS}\left(x\right)=\int_{0}^{x}\sin\left(t^{2}\right)dt \end{array},$$ [11]

where *γ*_1,2_ are magnetogyric ratios of the two electrons, and *r* is the inter-electron distance. All three operations are linear filters; the network managed to pack them into one layer. The function of the layer is now clear—baseline elimination, noise elimination, and signal preprocessing.

**Fig. 3.**
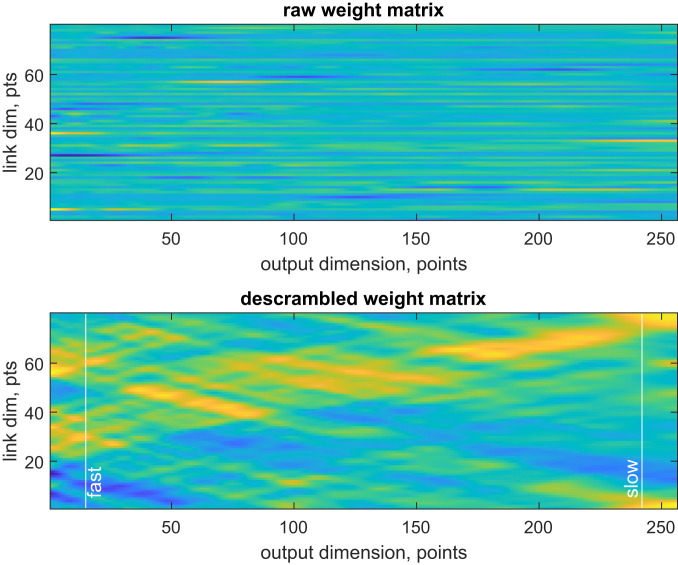
Spontaneous emergence of a time–distance transform in the weight matrix of the second fully connected layer of DEERNet (18). Once the descrambler group method is applied to the raw weight matrix (*Upper*), its vertical dimension becomes interpretable (*Lower*). The horizontal dimension here is the distance axis of the output—the matrix appears to map faster input oscillations (vertical line labeled “fast”) into shorter distances, and slower input oscillations (vertical line labeled “slow”) into longer distances. The detailed analysis of input and output singular vectors is performed in Fig. 4. Dim., dimension; pts., points.

Since the preceding layer is a digital filter that keeps the signal in the time domain, some form of time–distance transformation is expected in the weight matrix of the second fully connected layer (Fig. 3, *Upper*). Applying the descrambler group method to minimize simultaneously the second derivative norm of the output of the activation function of the previous layer, and the second derivative along the link dimension of **W**_2_, does indeed reveal a transformation (Fig. 3, *Lower*) that maps faster oscillations into shorter distances and slower oscillations into longer distances.

**Fig. 4.**
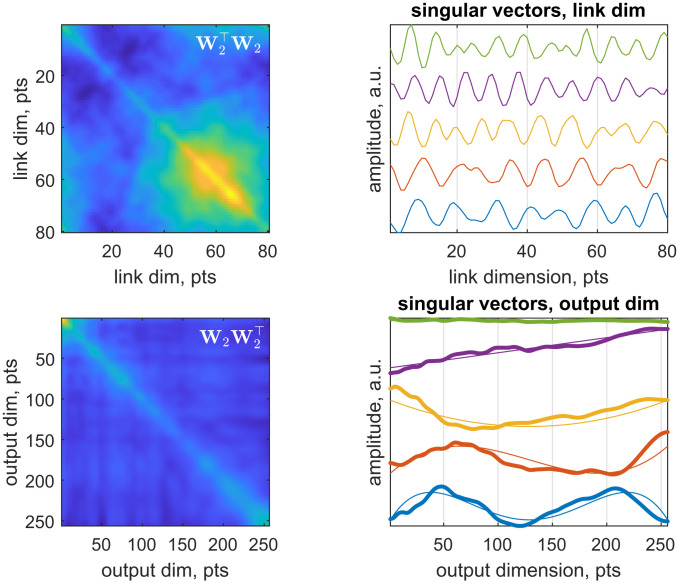
Spontaneous emergence of frequency-division multiplexing and Chebyshev polynomials in the second fully connected layer of a DEERNet (18) neural net. Descrambling the link dimension reveals an approximately orthogonal (*Upper Left*) conjugate signal library that SVD shows to be distorted sinusoids (*Upper Right*). The output signal library also appears to be approximately orthogonal (*Lower Left*); SVD reveals spontaneous emergence of distorted Chebyshev polynomials as the entries of that library (*Lower Right*). Dim., dimension; a.u., arbitrary units; pts., points.

A more detailed inspection reveals that both the rows and the columns of the descrambled **W**_2_ are approximately orthogonal (Fig. 4, *Upper Left* and *Lower Left*). This prompted us to run singular value decomposition (SVD) to find out what the descrambled weight matrix expects to receive and to send out. SVD is useful after descrambling because its structure

$$W=USV^{†},$$ [12]

naturally breaks the weight matrix down into an orthogonal set of conjugate signals that it expects to receive (columns of **V**), amplification coefficients for the signals received (elements of the diagonal matrix **S**), and an orthogonal set of signals that it expects to send out (columns of **U**) with those amplification coefficients in response to each of the signals it has received (24). SVD revealed that the conjugate input signals are sinusoids, slightly distorted, likely due to imperfect training (Fig. 4, *Upper Right*)—the network apparently invented frequency-division multiplexing. The output signals appear to be distorted Chebyshev polynomials (Fig. 4, *Lower Right*).

Exactly why the network went specifically for Chebyshev polynomials is unclear, but they provide the explanation of how regularization is done inside DEERNet: the ranks of the Chebyshev polynomials seen in the output signal library are smaller than the ranks that can, in principle, be digitized on the 256-point output grid. Thus, a degree of smoothness is enforced in the output signal—the procedure is reminiscent of spectral filtering regularization, which is also apparent in that the rank of the weight matrices is significantly smaller than the input dimension. This procedure has a modicum of elegance: the log-sigmoidal transfer function of the output layer in DEERNet neatly converts Chebyshev polynomials into patterns of peaks, as required by the physics of the problem (19). Importantly, SVD is only informative here after descrambling: singular vectors of a scrambled matrix are scrambled too.

The network is now completely interpreted: the first fully connected layer is a digital filter that performs denoising, baseline elimination, and frequency-axis rearrangement, and then sends the signal, in a frequency-multiplexed form, to the second fully connected layer, which performs a regularized time–distance transformation into Chebyshev polynomials that the final log-sigmoidal transfer function converts into the patterns of peaks seen in Fig. 1, *Right*.

To confirm the correctness of the DEERNet functionality interpretation, we have assembled a combination of digital filters that replicates the functionality of the first fully connected layer and a time–distance transformation that replicates the functionality of the second one.

**Fig. 5.**
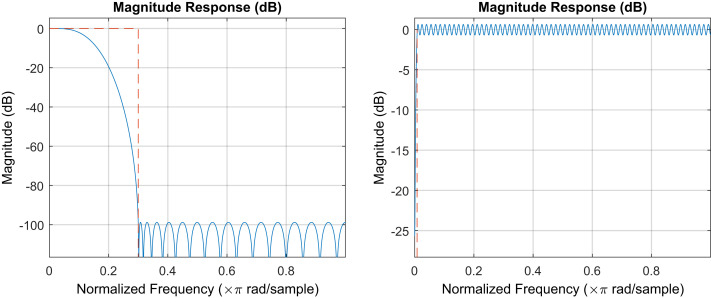
Digital filters used in the recreation of the functionality of the first fully connected layer of DEERNet. (*Left*) Notch filter at zero frequency, implemented as order 256 direct-form FIR high-pass filter with passband edge at 0.008 and stopband edge at 0.001 normalized frequency units. (*Right*) Order 32 direct-form FIR low-pass filter with passband edge at 0.01 and stopband edge at 0.3 normalized frequency units. Filters were created and analyzed by using the Signal Processing Toolbox of Matlab R2020a.

To emulate the first fully connected layer, we used standard finite impulse response (FIR) filters with pass and reject bands (Fig. 5) chosen to correspond approximately to the patterns seen in Fig. 2. The frequency axis rescaling transform and the regularized time–distance transform are both linear and were therefore combined into one matrix **T** that was obtained as a regularized pseudoinverse:

$$\begin{array}{c} T\left[\begin{array}{ccc} f_{1} & \ldots & f_{n} \end{array}\right]=\left[\begin{array}{ccc} p_{1} & \ldots & p_{n} \end{array}\right],n\gg \dim\left(T\right)\\ ⇓\\ {\left\|TF-P\right\|}_{\text{F}}^{2}+\lambda {\left\|T\right\|}_{\text{F}}^{2}=\min\\ ⇓\\ T={\left[{\left({\mathrm{FF}}^{\text{T}}+\lambda 1\right)}^{-1}\left({\mathrm{FP}}^{\text{T}}\right)\right]}^{\text{T}} \end{array}$$ [13]

where **p**_*k*_ are linearly independent-distance probability density distributions represented as vectors on a finite grid, and **f**_*n*_ are the corresponding solutions of Eq. **9**, also discretized on a finite grid. The regularization parameter *λ* was obtained using the L-curve method (17). Although some parameters (filter orders and bands, pseudoinverse regularization factor) were chosen empirically, they all now have a clear rational interpretation—thus, a physically meaningful data processing method was obtained from a descrambler group interpretation of a neural network.

**Fig. 6.**
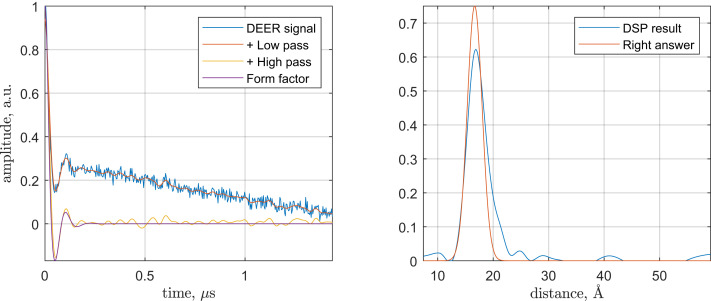
An example DEER processing run using the rational digital signal-processing replica of DEERNet. The calculation starts with a realistic randomly generated DEER dataset (*Left*; blue trace), for which the correct answer is known. The low-pass filter eliminates the noise (*Left*; red trace), and the notch filter at zero eliminates the baseline (*Left*; yellow trace). Up to the noise, the result matches the known right answer at this stage (*Left*; purple trace). The subsequent time–distance transform yields a distance pattern in reasonable agreement with the known right answer (*Right*). DSP, digital signal processing; a.u., arbitrary units.

The performance of the rationally constructed transform sequence is illustrated in Fig. 6—it is clear that the performance is similar to that shown by the neural network ensemble in Fig. 1. Across a large database of inputs that we had inspected, the rational method does require occasional pass and reject band adjustments in the digital filters to match the performance of the neural network, but those adjustments always have a physical explanation.

Further examples (a DEERNet with three fully connected layers and a network designed to eliminate additive noise from human voice recordings) may be found in *SI Appendix*. For the small networks analyzed in this work, descrambling results do not depend on the initialization—up to insignificant details (circular shifts in the descrambled link dimension, overall signs, phases of frequency components), we found the interpretation to be the same for each of the 100 independently initialized and trained nets that DEERNet is using for confidence interval estimation. It is possible that larger networks would differ in the strategies that they discover; we have not observed this yet.

## Conclusions and Outlook

The descrambler group method made it possible to interpret the functioning of a fully connected neural network. During its training, a simple DEERNet appears to have invented a bandpass filter, a notch filter, a frequency axis rescaling transformation, frequency-division multiplexing, spectral filtering regularization, and a map from harmonic functions into Chebyshev polynomials. As far as we can tell, a deeper DEERNet (*SI Appendix*, Section S4) also invented group embedding.

That these tiny networks should develop this amount of instantly recognizable mathematics and communications engineering in 10 min of unattended training from a random initial guess is unexpected. The functionality appears to be localized and readable to humans, meaning that reductionism (4) and critical rationalism (5) need not be abandoned, at least for the smaller neural networks. An ironic observation is that the act of interpreting the inner working of a static neural net apparently obviates the need for it: the same filters and transforms may now be applied rationally. It is also apparent that the number of effective parameters in the procedures that neural networks invent is much smaller than the raw number of network parameters; this agrees with the prior art (24–27).

A key strength of the descrambler group method is its applicability to fully connected layers—those are harder to interpret than convolutional layers, which inherit partial order from the convolution stride. Due to their importance in image processing, the existing interpretability scoring methods tend to focus on convolutional nets (28). Other established methods—for example, concept activation vectors (29) and saliency maps (30)—are specific to object detection and classification networks where identifiable concepts exist. This is not necessarily the case in digital signal processing networks like DEERNet that apply abstract nonlinear maps between vector spaces.

There is also a difference between finding out why an answer is produced and finding out how. The approach presented here is more firmly grounded in formal mathematics than many of the current explainable artificial intelligence techniques, of which some are variations of “poke it with a stick and see what happens” empiricism—that is what differentiating the network with respect to an input, output, or a parameter fundamentally is: increment something, look at the change in behavior. Much of the prior art deals specifically with expert, recommender, and classifier systems, and, thus, with extracting rule lists, decision trees, and taxonomies (31). It is sometimes possible, by very careful design reminiscent of the modeling used in physical sciences, to create classifier nets that are interpretable by construction (32). None of that is relevant to networks that evolve unknown mathematical transforms between abstract signal spaces—descrambler groups offer an opportunity here, because they run on generic mathematical properties of those signals. Descrambler groups also improve on the principal component analysis, which for weight matrices is essentially SVD. A scrambled weight matrix has scrambled singular vectors—only rank is then revealed by SVD; it can be informative (24), but only in the sense of telling the user to increase or reduce the layer dimension. However, after a descrambling transformation, singular vectors of fully connected layers become interpretable.

The definition of the descrambling target functional in general is entirely at the user’s discretion—the functional in Eq. **8** is only one of many possibilities. For example, in situations when frequency-domain data are expected at both the input and the output of an acoustic filter network, it is reasonable to seek a transformation of the intermediate signal space that makes intermediate signals maximally similar to the input ones. In that representation, the weight matrix **W** is expected to be diagonally dominant; this may be achieved by seeking an orthogonal transformation of the output space that achieves maximum diagonal sum or maximum diagonal norm square for the weight matrix:

$$\eta_{\text{MDS}}\left(Q\right)=\text{Tr}\left[PW\right]\;\eta_{\text{MDNS}}\left(Q\right)={\left\|\text{diag}\left(\mathrm{PW}\right)\right\|}_{2}^{2}.\;P=\frac{1-Q}{1+Q}$$ [14]

An example of using this approach for a network designed to remove additive noise from human speech is given in *SI Appendix*, section S5.

A discomfiting aspect of the present work is the amount of domain-specific expertise that was required to recognize the functionality of the descrambled weight matrices. It could be argued that the matrix in the second image of Fig. 2 is still uninterpretable to a nonspecialist. That is an improvement, though—the matrix in the first image was uninterpretable to everyone. We recommend a staged approach: the role of each layer should first be established empirically by using the prior art cited above, and then the weight matrix descrambled to find out the implementation details. The availability of such methods opens a way to deeper study of neural networks because the training stage can now be followed by the interpretation stage. So far, we have only seen our networks invent the mathematics that is known to humans. It is possible that, at some point, previously unknown mathematics would make an appearance: neural nets can likely be mined for new knowledge.

## Supplementary Material

Supplementary File

## Data Availability

The source code of DEERNet and its descrambler routines are available as a part of the open-source Spinach package (spindynamics.org).
