# Supplementary material for: Neural network interpretation using descrambler groups
Source: Proc Natl Acad Sci U S A. 2021 Jan 26;118(5):e2016917118. doi: 10.1073/pnas.2016917118 (PMC7865153; doi:10.1073/pnas.2016917118)
Supplement: Supplementary File [file pnas.2016917118.sapp.pdf]

Neural network interpretation using descrambler groups

Jake L. Amey, Jake Keeley, Tajwar Choudhury, and Ilya Kuprov*
University of Southampton, UK

Supplementary Information Appendix

S1. Gradient of the Tikhonov descrambling functional

The gradient of the functional in Equation (8) of the main text:

$$\eta_{\mathrm{T}}(\mathbf{Q}) = \left\| \mathbf{D}\frac{\mathbf{1}-\mathbf{Q}}{\mathbf{1}+\mathbf{Q}}\mathbf{W}_k \cdots F_1 \mathbf{W}_1 \mathbf{X} \right\|_{\mathrm{F}}^2 \tag{S.1}$$

may be obtained using matrix differentiation rules. The weighted array of column vector signals arriving from the preceding layers will be abbreviated as:

$$\mathbf{S} = \mathbf{W}_k F_{k-1} \mathbf{W}_{k-1} \cdots F_1 \mathbf{W}_1 \mathbf{X} \tag{S.2}$$

With the result that the descrambling functional at layer k becomes:

$$\eta_{\mathrm{T}}(\mathbf{Q}) = \left\| \mathbf{DPS} \right\|_{\mathrm{F}}^2 \qquad \mathbf{P} = \frac{\mathbf{1}-\mathbf{Q}}{\mathbf{1}+\mathbf{Q}} \tag{S.3}$$

where we chose **D** to be a second Fourier derivative (*1*) matrix. Using the chain rule:

$$\frac{\partial \eta_{\mathrm{T}}}{\partial Q_{ij}} = \mathrm{Tr}\left[\left(\frac{\partial \eta_{\mathrm{T}}}{\partial \mathbf{P}} \right)^{\mathrm{T}} \frac{\partial \mathbf{P}}{\partial Q_{ij}} \right] = \sum_{kl} \left[\frac{\partial \eta_{\mathrm{T}}}{\partial \mathbf{P}} \right]_{lk} \frac{\partial P_{lk}}{\partial Q_{ij}} \tag{S.4}$$

The derivative of **P** with respect to an element of **Q** is another instance of the chain rule:

$$\begin{aligned} \frac{\partial P_{lk}}{\partial Q_{ij}} &= \left[\frac{\partial}{\partial Q_{ij}} \left[(\mathbf{I}+\mathbf{Q})^{-1} (\mathbf{I}-\mathbf{Q}) \right] \right]_{lk} = \\ &= \left[\frac{\partial (\mathbf{I}+\mathbf{Q})^{-1}}{\partial Q_{ij}} (\mathbf{I}-\mathbf{Q}) + (\mathbf{I}+\mathbf{Q})^{-1} \frac{\partial (\mathbf{I}-\mathbf{Q})}{\partial Q_{ij}} \right]_{lk} = \\ &= \sum_m \frac{\partial \left[(\mathbf{I}+\mathbf{Q})^{-1} \right]_{lm}}{\partial Q_{ij}} [\mathbf{I}-\mathbf{Q}]_{mk} - \sum_n \left[(\mathbf{I}+\mathbf{Q})^{-1} \right]_{ln} \frac{\partial Q_{nk}}{\partial Q_{ij}} \end{aligned} \tag{S.5}$$

The last derivative is $\partial Q_{nk} / \partial Q_{ij} = \delta_{ni}\delta_{kj}$, and the derivative of the inverse matrix is:

www.pnas.org/cgi/doi/10.1073/pnas.2016917118

$$\frac{\partial\left[(\mathbf{I}+\mathbf{Q})^{-1}\right]_{lm}}{\partial Q_{ij}} = -\left[\left(\mathbf{I}+\mathbf{Q}\right)^{-1}\right]_{li}\left[\left(\mathbf{I}+\mathbf{Q}\right)^{-1}\right]_{jm} \tag{S.6}$$

This eliminates all derivatives and all explicit sums from the right-hand side:

$$\begin{aligned}\frac{\partial P_{lk}}{\partial Q_{ij}} &= -\sum_m\left[(\mathbf{I}+\mathbf{Q})^{-1}\right]_{li}\left[(\mathbf{I}+\mathbf{Q})^{-1}\right]_{jm}\left[\mathbf{I}-\mathbf{Q}\right]_{mk} - \sum_n\left[(\mathbf{I}+\mathbf{Q})^{-1}\right]_{ln}\delta_{ni}\delta_{kj} \\ &= -\left[(\mathbf{I}+\mathbf{Q})^{-1}\right]_{li}\left[(\mathbf{I}+\mathbf{Q})^{-1}(\mathbf{I}-\mathbf{Q})\right]_{jk} - \left[(\mathbf{I}+\mathbf{Q})^{-1}\right]_{li}\delta_{kj}\end{aligned} \tag{S.7}$$

Using the definitions of $\mathbf{P}$ and $\mathbf{I}$ yields further simplifications:

$$\frac{\partial P_{lk}}{\partial Q_{ij}} = -\left[(\mathbf{I}+\mathbf{Q})^{-1}\right]_{li}\left[\mathbf{I}+\mathbf{P}\right]_{jk} \tag{S.8}$$

Inserting this into Equation (S.4) produces:

$$\frac{\partial \eta_{\mathrm{T}}}{\partial Q_{ij}} = -\sum_{kl}\left[(\mathbf{I}+\mathbf{Q})^{-1}\right]_{li}\left[\frac{\partial \eta_{\mathrm{T}}}{\partial \mathbf{P}}\right]_{lk}\left[\mathbf{I}+\mathbf{P}\right]_{jk} \tag{S.9}$$

The explicit sum can now be collapsed:

$$\frac{\partial \eta_{\mathrm{T}}}{\partial Q_{ij}} = -\left[(\mathbf{I}+\mathbf{Q})^{-\mathrm{T}}\frac{\partial \eta_{\mathrm{T}}}{\partial \mathbf{P}}(\mathbf{I}+\mathbf{P})^{\mathrm{T}}\right]_{ij} \tag{S.10}$$

The derivative of η with respect to **P** is obtained using the Frobenius norm differentiation rule:

$$\frac{\partial \eta_{\mathrm{T}}}{\partial \mathbf{P}} = 2\mathbf{D}^{\mathrm{T}}\mathbf{DPSS}^{\mathrm{T}} \tag{S.11}$$

The final result is:

$$\frac{\partial \eta_{\mathrm{T}}}{\partial \mathbf{Q}} = -2(\mathbf{I}+\mathbf{Q})^{-\mathrm{T}}\left[\mathbf{D}^{\mathrm{T}}\mathbf{D}\right]\mathbf{P}\left[\mathbf{SS}^{\mathrm{T}}\right](\mathbf{I}+\mathbf{P})^{\mathrm{T}} \tag{S.12}$$

Numerical evaluation of both the functional and the gradient may be accelerated by pre-computing the terms enclosed in square brackets. Convergence criteria for training and descrambling must be sufficiently tight to suppress the numerical noise from the random numbers that are often used as the starting point in the neural network training process. Because smoothness is used as a criterion here, descrambling would fail if the intermediate signals cannot be made simultaneously smooth.

At the extremum, the descrambling functional in Equation (S.1) is stable with respect to small perturbations in $\mathbf{Q}$ by definition, because at extremum $\partial\eta_{\mathrm{T}}/\partial\mathbf{Q}=0$. Frobenius norm is submultiplicative, and therefore:

$$\left\|\mathbf{AB}\right\|_{\mathrm{F}} \le \left\|\mathbf{A}\right\|_{\mathrm{F}}\left\|\mathbf{B}\right\|_{\mathrm{F}} \quad \Rightarrow \quad \left\|\mathbf{D}\frac{\mathbf{1}-\mathbf{Q}}{\mathbf{1}+\mathbf{Q}}\mathbf{W}_k\cdots F_1\mathbf{W}_1\mathbf{X}\right\|_{\mathrm{F}} \le \alpha\left\|\mathbf{X}\right\|_{\mathrm{F}} \tag{S.13}$$

where α is a finite positive real number. Thus, η_{T} is also stable with respect to the input data: small variations in $\mathbf{X}$ are guaranteed to yield proportionally small variations in the interpretability score.

S2. Gradients of maximum diagonality descrambling functionals

The gradients of the two functionals (maximum diagonal sum and maximum diagonal norm squared) in Equation (14) of the main text

$$\eta_{\text{MDS}} = \text{Tr}\left[\mathbf{PW}\right], \qquad \eta_{\text{MDNS}} = \left\| \text{diag}\left(\mathbf{PW}\right) \right\|_2^2, \qquad \mathbf{P} = \frac{\mathbf{1}-\mathbf{Q}}{\mathbf{1}+\mathbf{Q}} \tag{S.14}$$

are obtained in a similar way to the above:

$$\begin{aligned} \frac{\partial \eta_{\text{MDS}}}{\partial \mathbf{Q}} &= -\left(\mathbf{1}+\mathbf{Q}\right)^{-\text{T}} \mathbf{W}^{\text{T}} \left(\mathbf{1}+\mathbf{P}\right)^{\text{T}} \\ \frac{\partial \eta_{\text{MDNS}}}{\partial \mathbf{Q}} &= -2\left(\mathbf{1}+\mathbf{Q}\right)^{-\text{T}} \left[\mathbf{W}^{\text{T}} \odot \text{diag}\left(\mathbf{PW}\right)\right] \left(\mathbf{1}+\mathbf{P}\right)^{\text{T}} \end{aligned} \tag{S.15}$$

where $\odot$ denotes element-wise multiplication.

S3. DEERNet topology and training

A training database containing 10^5 DEER traces was generated as we previously described (*2*); networks of the following general topology

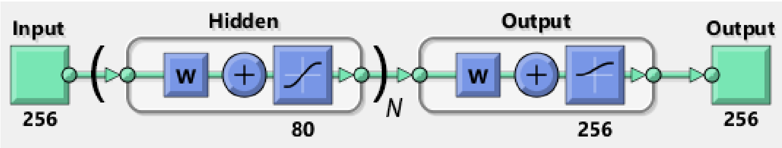

were trained using scaled conjugate gradient backpropagation on an NVidia Titan V card using Matlab R2020a Machine Learning Toolbox (*3*); technical details may be found in the same paper (*2*). The input layer and the inner layers of DEERNet use tangent sigmoidal activation functions, and the output layer uses the log-sigmoidal activation function to ensure that the output cannot become negative. The dimension of the inner layer weight matrices is chosen based on the weight matrix rank analysis (*2*).

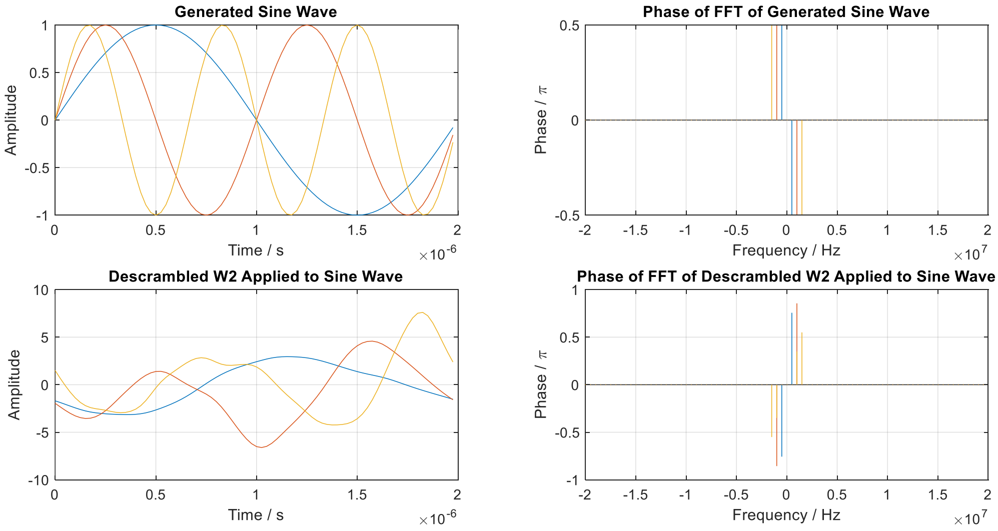

Figure S1. *Action by the descrambled form of the middle fully connected layer of DEERNet on a collection of sine waves.* ***Top left:*** *input signals (sine waves).* ***Top right:*** *input phases.* ***Bottom left:*** *output signals. Bottom right: output phases - note the inversion.*

S4. The extra layer of a deeper DEERNet

Descrambling the DEERNet featuring three fully connected layers revealed that the first layer applies a similar combination of digital filters to those shown in Figure 2 of the main text, and the last layer is a time-distance transform similar to the one in Figures 3 and 4. However, the middle fully connected layer turned out to have unexpected mathematical depth.

The descrambled weight matrix appears to be inverting the phases of the harmonics that it receives (Figure S3). However, this inversion is frequency-dependent: within the pass band of the digital filter in the previous fully connected layer, the matrix in Figure S4 is antidiagonal for some frequencies, but diagonal for others. Initially, this is puzzling – up to some tenacious noise that had survived the training, this corresponds to trivial phase rotations and inversions.

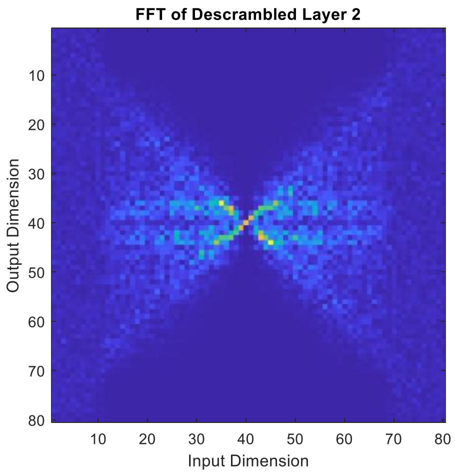

Figure S2. *Diagonal-antidiagonal structure of the Fourier transform of the descrambled middle layer weight matrix in the three-layer DEERNet.*

The essential need for phase inversions becomes clear once the group-theoretical nature of the DEER signal processing problem is considered. The integral transform in Eq (9) of the main text is, in some Euclidean metric, orthogonal. For input vectors of dimension n, the corresponding group is $O(n)$, and we are asking the network to approximate a particular element of this group. However, $O(n)$ is not a path-connected manifold because it has a discrete factor:

$$O(n) = SO(n) \otimes I \tag{S.16}$$

where $SO(n)$ is the special orthogonal group (which is path-connected), and I is the inversion group. Loosely speaking, there are two disconnected "copies" of $SO(n)$ in $O(n)$, and the transformation that the network needs to approximate would belong to one of these copies.

Consider the situation when the solution belongs to one instance of $SO(n)$, but the initial guess randomly drops the network into an approximation of the other instance. Because $O(n)$ is not path-connected, there is no way that a continuous function optimiser like gradient descent would be able jump from one copy to the other – unless the middle layer evolves an inversion component, which appears here to come embedded into a representation of $U(1)$ group of phase rotations. The network is apparently making use of the fact $SO(n) \otimes U(1)$ is path-connected, and that:

$$O(n) \subset SO(n) \otimes U(1) \qquad \text{(S.17)}$$

Thus the role of the middle fully connected layer in this larger DEERNet appears to be topological and group theoretical – the layer is a bridge between two disconnected components of the orthogonal group. The test of correctness of this hypothesis would be the sign of the determinant of the weight matrix – a direct inspection confirms that the determinant is negative.

S5. Descrambling the input layer of an acoustic filter network

A fully connected neural network designed to remove additive noise from recordings of human voice (*4*) is included as an example with the Deep Learning Toolbox of Matlab 2020a (*3*). Its training set comes from the Common Voice database (*5*). With the exception of bias vectors (which we removed from the network architecture before training to facilitate subsequent descrambling), the network was trained as described in Matlab R2020a documentation (*3*).

The input a [129 frequency points]×[8 time points] block of the short-time Fourier transform of the input signal, stretched column-wise into a 1032-element vector. Because the network receives frequency domain data, its input and intermediate signals are not expected to be smooth – the descrambling target functional must be different from the one used for DEERNet.

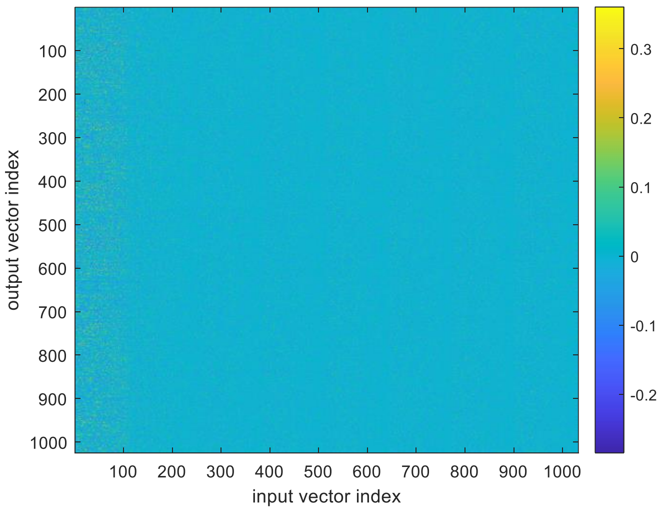

Figure S3. *First fully connected layer weight matrix of our acoustic filter network, showing the characteristic absence of directly visible structure.*

The immediate appearance of the weight matrix has no identifiable characteristics (Figure S3). This is typical for fully connected layers. However, the input dimension is expected to have a block structure matching the eight blocks of the input vector. This is visible in the row element autocorrelation function (Figure S4), which suggests a faint repeating pattern of 129 elements.

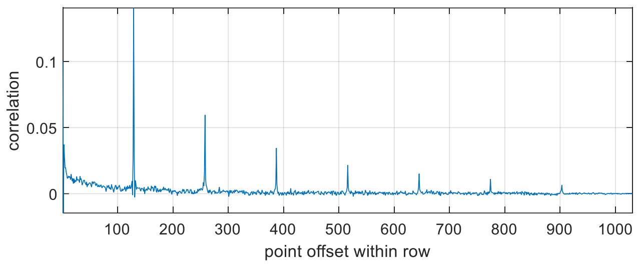

__Figure S4.__ The average (over all rows) autocorrelation function of the row elements of the weight matrix of the first fully connected layer of the acoustic filter network.

The inspection of the weight matrix average over the eight correlated blocks reveals an elaborate frequency filter (Figure S5): some input frequencies are mostly rejected, and linear combinations of others are sent to the output. This suggests that the function of the matrix is to attenuate undesired frequencies, meaning that a maximum diagonality descrambler (Section S2) would be appropriate here.

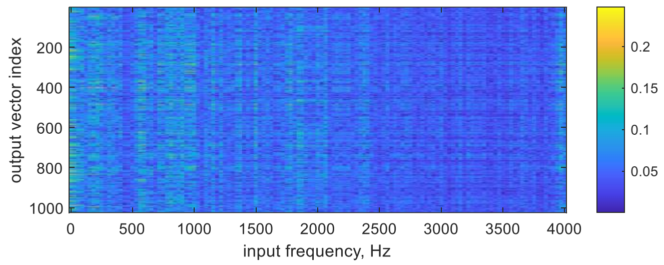

__Figure S5.__ Input vector block average of the acoustic filter network input layer weight matrix with insignificant singular values filtered out.

Descrambling using MDNS criterion reveals that selective frequency attenuation is not the only thing this layer does – only about 50% of the Frobenius norm of the weight matrix in Figure S3 is condensed to the diagonal by an orthogonal transformation acting on the output side (Figure S6, left panel). The rest of the norm accumulates on the diagonals of other STFT blocks (Figure S6, right panel).

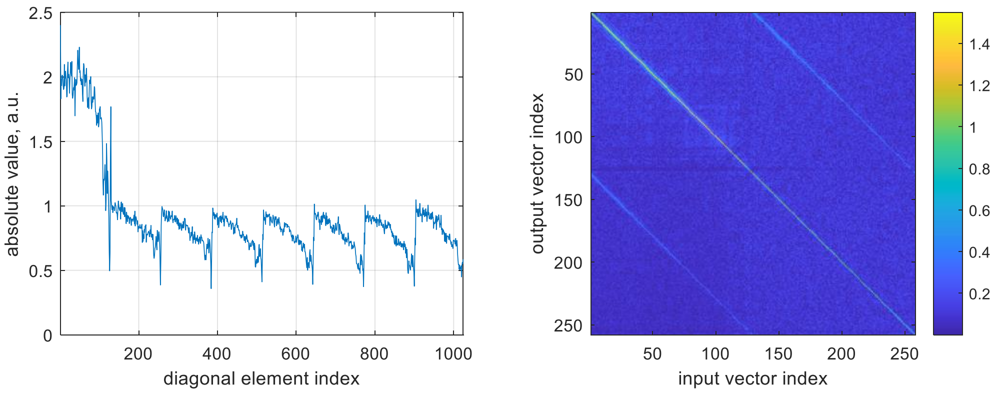

__Figure S6.__ Left panel: diagonal elements of the acoustic filter network input layer after descrambling using maximum diagonality criterion. Right panel: a zoom into the top left quarter of the descrambled weight matrix.

Thus, the descrambled weight matrix is multi-diagonal: the left panel of Figure S6 reveals that the main diagonal is applying a soft low pass filter to every STFT block, and the presence of other diagonals appears – at least algebraically – to correspond to a weighed averaging and/or cancellation of individual frequencies between the blocks. The exact nature of what is happening here will be of interest to DSP

specialists, this paper is not the place to investigate further. The salient point is that descrambling has again revealed interpretable structure in what previously (Figure S3) looked like noise.

S6. References

1. L. N. Trefethen, *Spectral methods in MATLAB*. (SIAM, 2000).
2. S. G. Worswick, J. A. Spencer, G. Jeschke, I. Kuprov, Deep neural network processing of DEER data. *Science Advances* **4**, eaat5218 (2018).
3. Matlab-R2020a. (The MathWorks Inc., Natick, Massachusetts).
4. D. Liu, P. Smaragdis, M. Kim, in *Fifteenth Annual Conference of the International Speech Communication Association*. (2014).
5. R. Ardila *et al.*, Common Voice: A Massively-Multilingual Speech Corpus. *arXiv preprint arXiv:1912.06670*, (2019).
